# Mutant Native Outer Membrane Vesicles Combined with a Serogroup A Polysaccharide Conjugate Vaccine for Prevention of Meningococcal Epidemics in Africa

**DOI:** 10.1371/journal.pone.0066536

**Published:** 2013-06-21

**Authors:** Rolando Pajon, Andrew M. Fergus, Dan M. Granoff

**Affiliations:** Center for Immunobiology and Vaccine Development, Children’s Hospital Oakland Research Institute, Oakland, California, United States of America; University of Cambridge, United Kingdom

## Abstract

**Background:**

The meningococcal serogroup A (MenA) polysaccharide conjugate vaccine used in Sub-Saharan Africa does not prevent disease caused by MenW or MenX strains, which also cause epidemics in the region. We investigated the vaccine-potential of native outer membrane vesicles with over-expressed factor H-binding protein (NOMV-fHbp), which targeted antigens in African meningococcal strains, and was combined with a MenA polysaccharide conjugate vaccine.

**Methodology/Principal Findings:**

The NOMV-fHbp vaccine was prepared from a mutant African MenW strain with PorA P1.5,2, attenuated endotoxin (ΔLpxL1), deleted capsular genes, and over-expressed fHbp in variant group 1. The NOMV-fHbp was adsorbed with Al(OH)_3_ and used to reconstitute a lyophilized MenA conjugate vaccine, which normally is reconstituted with liquid MenC, Y and W conjugates in a meningococcal quadrivalent conjugate vaccine (MCV4-CRM, Novartis). Mice immunized with the NOMV-fHbp vaccine alone developed serum bactericidal (human complement) activity against 13 of 15 African MenA strains tested; 10 of 10 African MenX strains, 7 of 7 African MenW strains, and 6 of 6 genetically diverse MenB strains with fHbp variant group 1 (including 1 strain from The Gambia). The combination NOMV-fHbp/MenA conjugate vaccine elicited high serum bactericidal titers against the two MenA strains tested that were resistant to bactericidal antibodies elicited by the NOMV-fHbp alone; the combination elicited higher titers against the MenA and MenW strains than those elicited by a control MCV4-CRM vaccine (P<0.05); and high titers against MenX and MenB strains. For most strains, the titers elicited by a control NOMV-fHbp knock out vaccine were <1∶10 except when the strain PorA matched the vaccine (titers >1∶000).

**Conclusion/Significance:**

The NOMV-fHbp/MenA conjugate vaccine provided similar or higher coverage against MenA and MenW strains than a quadrivalent meningococcal conjugate vaccine, and extended protection against MenX strains responsible for epidemics in Africa, and MenB strains with fHbp in variant group 1.

## Introduction

Devastating epidemics of meningococcal disease have occurred in sub-Saharan Africa for over one hundred years [1]. In 1996, 25,000 deaths were reported, which was the largest meningococcal epidemic on record. Most meningococcal disease in sub-Saharan Africa is caused by serogroup A (MenA) strains. In contrast, in industrialized countries nearly all meningococcal disease is caused by strains producing capsular serogroups B, C or Y [2]–[4]. Until recently, control of meningococcal epidemics in Africa remained an unsolved public health challenge. After more than ten years of work [5]–[7] a MenA polysaccharide-protein conjugate vaccine (MenAfriVac) was introduced [8]–[10]. As of December 3^rd^ 2012, 100 million people in sub-Sahara had received the vaccine (http://www.path.org/news/pr121203-menafrivac.php). Early data indicated that vaccination has been effective in both preventing MenA meningococcal disease [11] and decreasing colonization by MenA strains [12]. Thus, this vaccine has the potential to eliminate MenA epidemics [11]. Widespread vaccination, however, could allow emergence of strains with other serogroups such as X (MenX) or W (MenW), which also cause epidemics in this region [13]–[15]. Although it is not easy to determine whether the vaccination campaign has affected disease caused by strains with non-vaccine serogroups, in Burkina Faso, which was one of the first countries to use the MenA conjugate vaccine, more than 5000 cases and 550 deaths caused by MenW isolates were reported during the first 5 months of 2012 (WHO Surveillance Bulletins, http://www.who.int/csr/don/2012_05_24/en/index.html).

Commercial quadrivalent meningococcal A,C,Y and W conjugate vaccines are available in industrialized countries [16]–[19]. While these vaccines could prevent MenA or MenW disease in sub-Sahara, the vaccines are not affordable since this region is one of the poorest in the world. Also quadrivalent vaccines do not prevent MenX disease, or disease caused by strains with serogroup B (MenB), which are responsible for meningococcal disease in industrialized countries and could arise in Africa in the future.

Development of polysaccharide-protein conjugate vaccines for prevention of MenB disease has not been possible because the MenB polysaccharide is an autoantigen [20]. Newly discovered protein antigens are under investigation as vaccines that target MenB disease. One of the most promising is factor H-binding protein (fHbp) [21], [22], which is an important virulence determinant [23], [24], and is expressed by nearly all strains [25], [26]. fHbp is contained in two meningococcal B vaccines [27]–[31]. Recently one of these vaccines (Bexsero, Novartis Vaccines, Siena Italy), was approved by the European Commission for immunization of individuals two months of age and older [32]. Second-generation vaccines using chimeric fHbp [33], [34], new adjuvants [35], or native (non-detergent treated) outer membrane vesicle (NOMV) vaccines from mutants with genetically attenuated endotoxin and over-expressed fHbp (NOMV-fHbp) [36], [37], also are in development, and offer the prospect of enhancing immunogenicity and breadth of protection.

More than 600 different fHbp amino acid sequence variants have been identified (http://pubmlst.org/neisseria/fHbp/). Each one is given a unique identifier (ID number). Based on amino acid relatedness, fHbp sequences have been classified into two sub-families (A or B) or three variant groups (v.1, v.2, or v.3) [21], [22]. In general, serum antibodies elicited by fHbp vaccines have bactericidal activity only against strains with fHbp in the same variant group or sub-family as the vaccine [21], [22], [27], [28], [30], [31], [33], [34], [38]–[41].

In a recent study, we immunized mice with a prototype NOMV-fHbp vaccine prepared from a mutant MenB strain over-expressing fHbp ID 1, which is a prevalent variant group 1 sequence among MenB strains in the United States [26]. The animals developed serum bactericidal antibody responses against MenB strains and also against epidemic MenA, W and X strains from sub-Saharan Africa [42]. While these results were promising, neither the PorA variable region (VR) type of the mutant group B strain used to prepare the NOMV vaccine, or the fHbp ID, were present among African meningococcal strains. Also, we had used a plasmid construct to over-express fHbp in the mutant vaccine strain. As a consequence the resulting mutant strain would be too unstable for use in a commercial vaccine production. In the present study we used an engineered promoter containing neisserial *porA* and *nadA* promoter sequences, which integrated into the bacterial chromosome and provided stable over-expression of fHbp. Mutant strains with this promoter are potentially suitable for commercial NOMV vaccine production. Further the mutant strain incorporated new knowledge about the advantages of using non-fH binding fHbp mutants as vaccine antigens [43]–[45], which were gained from studies of MenB vaccines, into a single prototype strain designed specifically to target antigens in epidemic meningococcal strains from sub-Sahara Africa. Because our long term goal is a vaccine intended to extend protection elicited by the MenA conjugate vaccine being used in sub-Sahara to cover MenW and X strains, we also investigated the feasibility of combining the NOMV-fHbp vaccine with a MenA polysaccharide-protein conjugate vaccine.

## Methods

### Objectives

The primary objective was to investigate the immunogenicity in mice of a prototype meningococcal NOMV-fHbp vaccine prepared from a mutant strain from Africa engineered to over-express fHbp when combined with a MenA polysaccharide-protein conjugate vaccine. Our hypothesis was that the combination NOMV-fHbp/MenA conjugate vaccine would elicit similar or higher serum bactericidal antibody responses against epidemic meningococcal MenA, W or X strains than a U.S./European-licensed quadrivalent meningococcal polysaccharide-protein conjugate vaccine.

### Descriptions of Procedures

#### Selection of fHbp sequence variant and vaccine strain

We previously identified four prevalent fHbp amino acid sequence variants among a collection of 106 MenA, W and X meningococcal isolates from 17 countries in Africa [42] (see also Figure S1). These four fHbp sequence variants (including related variants, each differing from the respective prevalent variant by 1 amino acid) were present in 81% of the isolates. With the exception of fHbp ID 22/23 in variant group 2 (which was in 58% of MenW isolates), the three other prevalent fHbp sequence variants were in variant group 1∶100% of MenA isolates, 95% of MenX isolates, and 34% of MenW isolates. For over-expression of fHbp, we selected fHbp ID 9 as potentially the most cross-reactive protein among the prevalent variant group 1 proteins [42]. The amino acid sequence of fHbp ID 9 was 96 and 94 percent identical to ID 4/5 or 74, respectively, and was predicted to elicit protective anti-fHbp antibodies against MenA, W and X strains with fHbp in variant group 1. For coverage against MenW strains with fHbp variant 2, we selected an invasive MenW strain (Sudan 1/06), to prepare the mutant vaccine strain. The PorA (P1.5,2) of this strain was identical to that of all epidemic ST-11 MenW strains tested from sub-Saharan Africa with fHbp in variant groups 1, 2, or 3. This particular PorA subtype also was related to P1.5–1,2–36, and P1.5–1,2–2, which are found in clonal complex (CC) 175 MenW strains that cause disease in the sub-Sahara but are less common than CC11 MenW strains. The PorA P1.2,5 in vaccine also was antigenically related to PorA 5-1,10-1, which are present in recent CC181 MenX strains [42], [46]. Thus, the PorA from the vaccine strain was predicted to elicit broadly protective anti-PorA antibodies against the majority of MenW isolates and, possibly, against some MenX strains from the sub-Saharan region irrespective of the fHbp sequence variant.

#### Stable over-expression of fHbp ID 9

**Figure 1:**
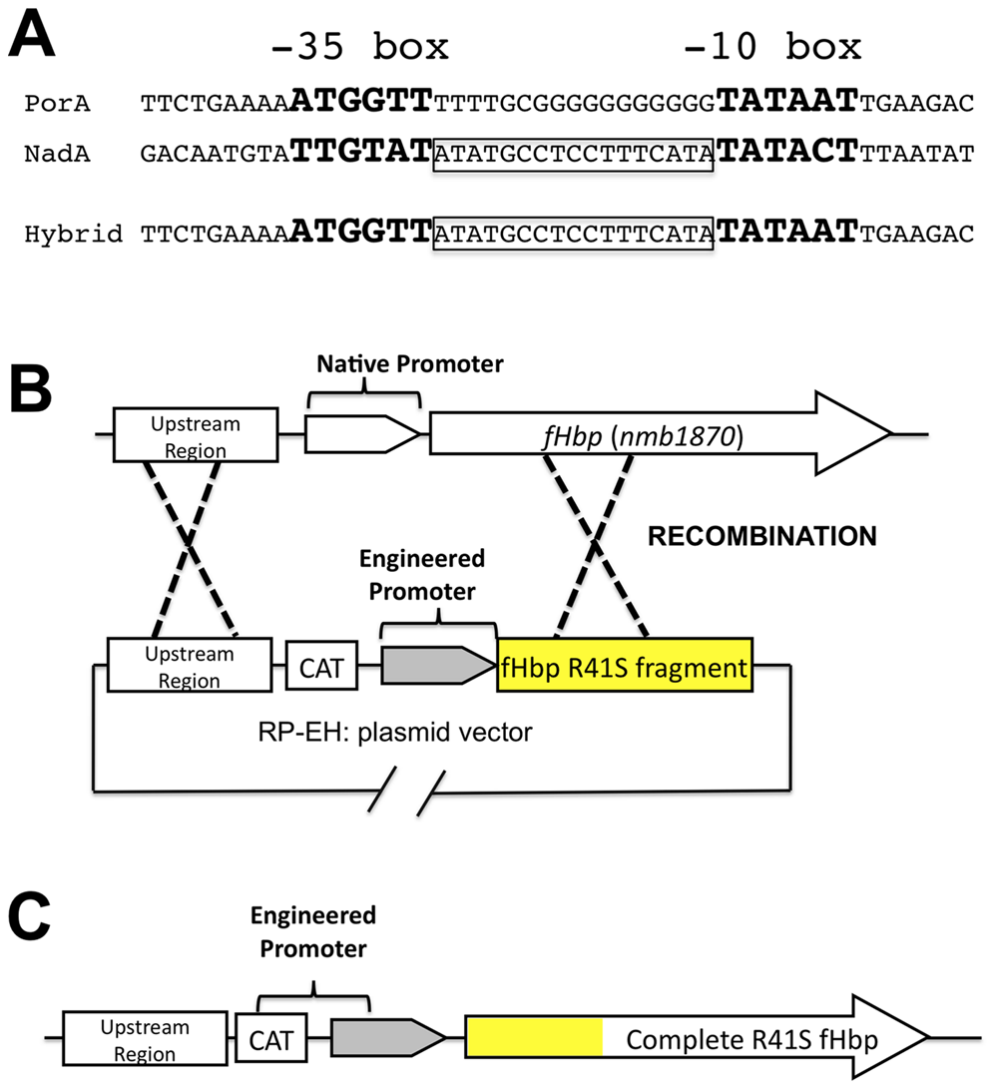
Schematic representation of the genetic strategy to over-express fHbp ID 9 R41S. Panel A: Sequences of natural *porA* and *nadA* promoters and the engineered (Hybrid) promoter where the sequence between the −10 and −35 boxes of the *porA* promoter containing the poly G tract was replaced with the sequence from the Neisserial *nadA* promoter to eliminate slip-strand miss-pairing. Although not shown, in the hybrid promoter the *porA* promoter sequence (−164 to −35 and −10 to +57) was left intact. Panels B-C: Strategy for replacement of the native fHbp promoter by the engineered promoter sequence, and introduction of the R41S mutation in fHbp to decrease fH binding (see text for additional details). Panel B shows the genomic environment of the fHbp gene (*nmb1870*) and the plasmid RP-EH used. CAT, represents the chloramphenicol resistance cassette used for selection of the recombinants. The yellow R41S box represents the first 255 base pairs of the fHbp ID 9 gene containing the mutation that leads to the R41S change in the mature mutant ID 9 fHbp. Panel C represents the organization of the region after the recombination.

Strain Sudan 1/06 is naturally a low-to-medium expresser of fHbp ID 9 [42]. For over-expression of fHbp we replaced the native fHbp promoter with an engineered hybrid *nadA-porA* promoter (Figure 1, Panel A). In the engineered promoter, designated EH, the sequence between −10 and −35 in the PorA promoter that contained the poly G tract, which promotes slip-strand miss-pairing, was replaced with the sequence (ATATGCCTCCTTTCATA) from the neisserial *nadA* promoter to stabilize expression of the downstream gene (fHbp, *nmb1870*, Figure 1, Panel B).

**Figure 2:**
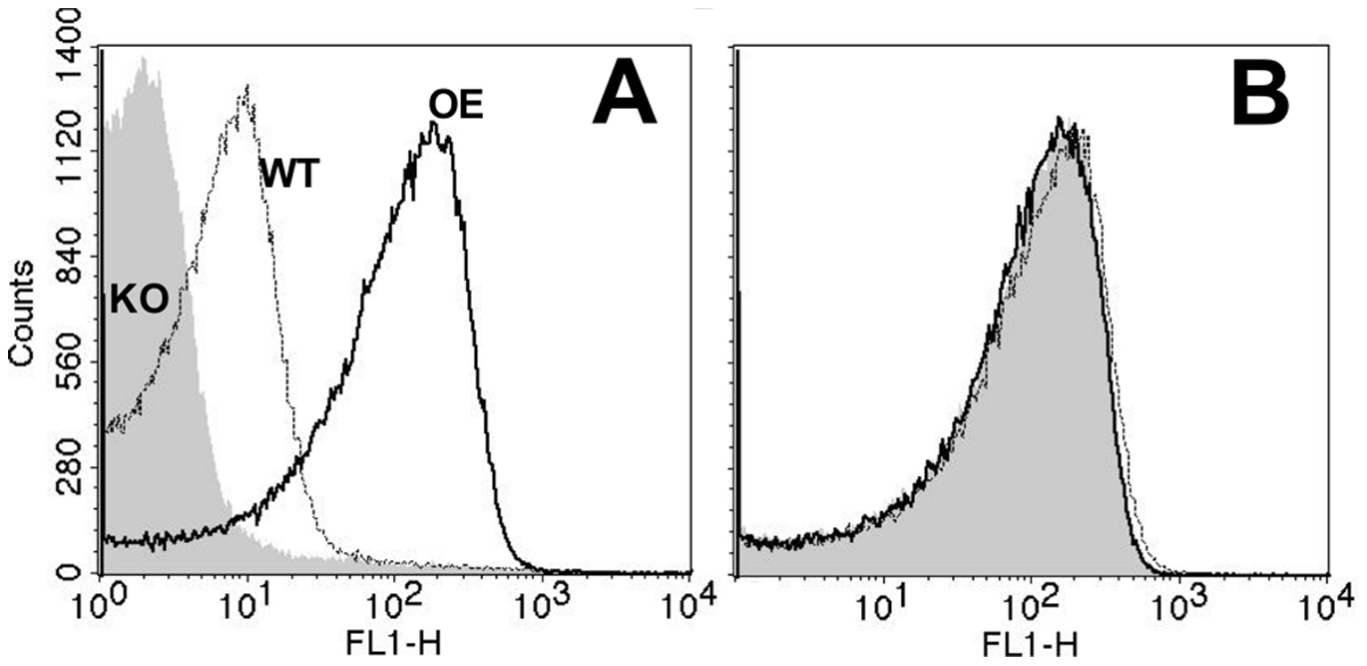
Surface-accessible fHbp on live bacteria of the mutant vaccine strain Sudan 1/06 with over-expressed fHbp as measured by flow cytometry. A. Binding of anti-fHbp mAb, JAR 5. WT, parental strain with wildtype fHbp expression; KO, mutant with fHbp gene inactivated; OE, strain with the engineered promoter and mutant gene encoding fHbp ID 9 with the R41S amino acid substitution. Panel B: Detection of MenW using anticapsular mAb JW-W1 as a control.

In previous studies, fHbp vaccines with an R41S mutation had decreased fH binding and showed increased immunogenicity in human fH transgenic mice than control fHbp vaccines that bound human fH [43], [44]. In the present study, we introduced the R41S mutation into a fragment containing the first 255 base pairs of the fHbp gene, which is a highly conserved region [38], [47] among the variant group 1 fHbp sequences and was used as the 3′ recombination arm. The engineered EH promoter and the plasmid vector, which contains the promoter and the 255 base pairs of the fHbp gene including the mutation encoding for R41S, are illustrated in Figure 1, Panels B and C, respectively. Using this promoter we generated a mutant of strain Sudan 1/06, designated OE fHbp (OE, over-expressed), and used flow cytometry with anti-fHbp mAb JAR 5 to assess the relative amounts of fHbp on the bacterial surface of live bacteria [48]. The mutant strain with over-expressed fHbp R41S had ∼10-fold increased fHbp on its surface compared with the parent wildtype strain (Figure 2).

For the final vaccine strain we inserted a second copy of the engineered promoter, the R41S fHbp gene, a terminator, and a different antibiotic marker, neomycin phosphotransferase, that confers kanamycin resistance, into the *lpxL1* locus to generate a mutant with two copies of the R41S fHbp and penta-acylated LOS instead of hexa-acylated LOS in the parental strain [49]. Penta-acylated LOS has 50- to 100-fold diminished endotoxin activity compared to that of wildtype hexa-acylated LOS [49]–[51] (See Discussion section). The second copy of the engineered promoter/fHbp gene also increased fHbp levels in the NOMV by ∼25% over those achieved by a single copy in the native fHbp locus (data not shown). Finally, to create a capsular null mutant, we deleted a chromosomal segment (*cssA-cssE_w_*) by recombining a cassette that conferred erythromycin resistance to enable selection of the recombinants. This mutation eliminated the MenW polysaccharide in the NOMV vaccine, avoided the potential problem of induction of anti-capsular antibody hyporesponsiveness [52], [53], and helped ensure production of a consistent NOMV vaccine.

As described below, the resultant capsular null mutant vaccine strain with attenuated endotoxin and over-expressed R41S fHbp was used to produce the NOMV-fHbp vaccine. We created a second mutant strain with the same modifications and antibiotic resistance genes but lacking the fHbp gene (NOMV-fHbp KO), which was used to produce a control NOMV vaccine without fHbp (NOMV-KO). The results of characterization of the different NOMV vaccines are provided in the Results section.

#### Preparation of NOMV vaccines

Bacteria were grown at 37°C with shaking at 300 rpm for 10 hours in a 600 mL culture in Frantz media to early stationary phase (OD_600 nm_ 3.8 to 4.2 after 10 hours of growth). Phenol 0.5% (w/v) was added to inactivate the bacteria. After overnight incubation at 4°C, the culture was centrifuged for 40 minutes at 4000 x g and the supernatant was filtered through a 0.45 µM Millipore filter unit (PES, 500ml, 90 mm diameter, Thermo Fisher Scientific, MA, US) to remove remaining bacteria. The supernatant was concentrated to ∼100 ml in an ultrafiltration cell (Millipore Model 8400, Millipore Ultrafiltration Membranes, PLHK, 76 mm diameter, 100,000 NMWL, Millipore, MA, US), and centrifuged at 100,000 g for 2 hours (41,100 rpm, Ti60 rotor, Beckman ultracentrifuge). The NOMV blebs were re-suspended in 3% sucrose and 0.2 M glycine, pH 8.0, and stored at –20°C.

#### NOMV characterization

Major proteins in the NOMV vaccines were separated by SDS PAGE and visualized with Commassie Blue staining (see Figure S2). The concentration of fHbp in the NOMV was measured by a quantitative Western blot, which had a linear range between 0.1 to 2 mg/ml [38]. The anti-fHbp mAb used was JAR 5 [54]. The presence of fHbp, PorA, MenW capsule, and residual fH-binding in the NOMV were measured by ELISA, which was performed as previously described [43].

#### Mouse immunization

CD-1 female mice were obtained from Charles River (Wilmington, MA, US). Groups of five-week-old mice (12 mice per group) were immunized with NOMV-fHbp or NOMV-KO vaccines (2.5 µg of total protein). As an additional control, we immunized mice with a quadrivalent meningococcal conjugate vaccine (Menveo, Novartis), which was designated “meningococcal conjugate vaccine 4-CRM,” or MCV4-CRM. Each human dose of MCV4-CRM contains 10 µg of MenA oligosaccharide and 5 µg each of oligosaccharides of MenC, Y and W conjugated with CRM_197_ protein. The commercial vaccine is supplied in two vials, one containing lyophilized MenA conjugate, which is reconstituted immediately before use with liquid MenC, Y and W conjugate vaccines in the second vial. The vaccine for humans does not use an aluminum adjuvant. For the mouse immunogenicity study, the vaccine was adsorbed with 0.6 mg of aluminum hydroxide (2% Alhydrogel, Brenntag Biosector), and we used one-fifth of the human dose (i.e., for mice, 1 µg of each of the MenC, Y and W oligosaccharide conjugates and 2 µg of the MenA conjugate). The NOMV vaccines were also adsorbed with aluminum hydroxide (0.6 mg per dose). For the combination vaccine, we reconstituted 2 µg of lyophilized MenA conjugate vaccine with the liquid suspension of NOMV-fHbp vaccine (2.5 µg of protein) that had been adsorbed with aluminum hydroxide. Three injections were given, each separated by 3 weeks, and terminal blood samples were obtained three weeks after the last dose.

#### Measurement of serum antibody responses by ELISA

Serum IgG anti-fHbp Ab responses were measured by ELISA, which was performed as described previously [45]. Serum anticapsular antibody responses were measured using MenA polysaccharide coupled to adipic acid dihydrazide [55]. For coating the plates we used 5 µg/mL of the derivatized polysaccharide.

#### Serum bactericidal antibody activity

Serum bactericidal titers were measured as previously described using early log-phase bacteria grown for approximately 2 h in Mueller-Hinton broth (BD Biosciences, Franklin Lakes, NJ, US) supplemented with 0.25% glucose (w/v) and 0.02 mM cytidine 5′-monophospho-N-acetylneuraminic acid (CMP-NANA, Sigma-Aldrich, St, Louis, MO, US). To insure that all of the test strains expressed sialylated LOS, we added CMP-NANA to the culture media since some *N. meninigitidis* strains (particularly serogroup A) require exogenous CMP-NANA for sialylation of LOS while others have mechanism(s) for endogenous sialylation [56], [57]. Strains with sialylated LOS are more resistant to bactericidal activity than strains with no or partial sialylated LOS [58]. Therefore, the titers reported are conservative estimates of protective activity. The complement source was IgG-depleted human serum, which was prepared as previously described [38]. We tested serum bactericidal activity against 38 strains (15 MenA, 6 MenB, 7 MenW and 10 MenX). Source, phenotypic and genetic characterization on the strains [42], [59]–[61] are summarized in Table 1.

**Table 1:** Summary of meningococcal strains used to test serum bactericidal activity.

Strain Name	Secondary Strain Designation	Capsular Group	Country	Year	fHbp Protein ID*	1/Serum Bactericidal GMT†		Clonal Complex, (ST)	PorA VR Type	Reference
						NOMV-KO	NOMV-fHbp			
Senegal1/99	A1	A	Senegal	1999	5	<10	281	5 (5)	P1.20,9	[59]
BuFa6/07	A2	A	Burkina Faso	2007	5	16	108	5 (2859)	P1.20,9	[59]
E23/03	A3	A	Ethiopia	2003	5	<10	<10	5 (7)	P1.20,9	[59]
Z1275	A4	A	Niger	1963	4	<10	100	1 (1)	P1.5–2,10	[60]
E2/88	A5	A	Ethiopia	2003	5	<10	346	5 (7)	P1.20,9	[59]
Z2491	A6	A	Gambia	1983	5	<10	6861	4 (4)	P1.7,13–1	[61]
Niger1/95	A7	A	Niger	1995	5	31	463	5 (5)	P1.20,9	[59]
Niga3/07	A8	A	Nigeria	2007	5	<10	178	5 (7)	P1.20,9	[59]
C50/01	A9	A	Congo	2001	5	<10	<10	5 (7)	P1.20,9	[42]
LNP20868	A10	A	Burkina Faso	2003	5	<10	306	5 (2859)	P1.20,9	[59]
LNP20790	A11	A	Burkina Faso	2003	5	<10	71	5 (2859)	P1.20,9	[59]
Nigeria4/03	A12	A	Nigeria	2003	5	632	1643	5 (7)	P1.20,9	[42]
Sudan11/07	A13	A	Sudan	2007	5	<10	109	5 (7)	P1.20,9	[42]
BuFa20030378	A14	A	Burkina Faso	2007	5	<10	160	5 (6035)	P1.20,9	[42]
Chad5/07	A15	A	Chad	2007	5	<10	183	5 (7)	P1.20,9	[42]
H44/76	B1	B	Norway	1976	1	<10	736	32 (32)	P1.7,16	[42]
SK140	B2	B	US	2005	4	<10	1151	41/44 (5097)	P1.7–2,4	[42]
MD01327	B3	B	US	1999	4	<10	893	41/44 (2973)	P1.18,25	[42]
NZ98/254	B4	B	New Zealand	1998	14	<10	368	41/44 (42)	P1.7–2,4	[42]
M01573	B5	B	US	NA	55	<10	125	41/44 (44)	P1.7–1,1	[22]
675	B6	B	The Gambia	1995	347	<10	3065	41/44 (8233)	P1.7–1,1	[42]
††Su1/06	W1	W	Sudan	2006	9	23280	25411	11 (11)	P1.5,2	[59]
BuFa2-03	W2	W	Burkina Faso	2003	23**	2014	1243	11 (11)	P1.5,2	[59]
Nigeria1/04	W3	W	Nigeria	2004	22**	87	34	175 (2881)	P1.5–1,2–36	[42]
C6/01	W4	W	Congo	2001	350	221	1753	175 (175)	P1.5–1,2–2	[42]
BF 17/01	W5	W	Burkina Faso	2001	9	1374	1294	11 (11)	P1.5,2	[42]
Uganda12/06	W6	W	Uganda	2006	349	1533	781	11 (11)	P1.5,2	[42]
Mali1/06	W7	W	Mali	2006	23**	1309	753	11 (11)	P1.5,2	[42]
BF 12/03	X1	X	Burkina Faso	2003	73	38	1627	181 (751)	P1.5–1,10–1	[42]
Kenya1/06	X2	X	Kenya	2006	74	<10	310	UA (5403)	P1.19,26	[42]
Uganda 23/06	X3	X	Uganda	2006	74	<10	277	UA (5403)	P1.19,26	[42]
Uganda 3/06	X4	X	Uganda	2006	74	<10	112	UA (5403)	P1.19,26	[42]
Uganda14/06	X5	X	Uganda	2006	74	<10	118	UA (5403)	P1.19,26–4	[42]
BF 5/97	X6	X	Burkina Faso	1997	73	<10	239	181 (751)	P1.5–1,10–1	[42]
BuFa2-97	X7	X	Burkina Faso	1997	73	<10	446	181 (751)	P1.5–1,10–1	[59]
BuFa7/07	X8	X	Burkina Faso	2007	74	93	279	181 (181)	P1.5–1,10–1	[59]
HF24	X9	X	S. Africa	1970s	4	21	160	181 (3687)	P1.7,9	[42]
HF78	X10	X	S. Africa	1970s	4	11	359	181 (3687)	P1.7,9	[42]

* As designated on the public website at http://pubmlst.org/neisseria/fHbp/. **fHbp sequence variant in group 2 (sub-family A); all other fHbp sequence variants are in group 1 (sub-family B) as designated on the public website.

† For strains A01–A05, W01–W04, X01–X03, and B01–B05, the reciprocal GMT was calculated from titers of three serum pools (four animal each). These are shown graphically along with GMT of negative control mice immunized with aluminum hydroxide alone in Figure 5. For the remaining strains shown in Table 1, the reciprocal GMT was calculated from titers of two serum pools.

†† Strain used for construction of the vaccine strain.

¶ In this publication we made use of the Neisseria Multi Locus Sequence Typing website (http://pubmlst.org/neisseria/) developed by Keith Jolley and sited at the University of Oxford [93], [94].

#### Statistical analyses

Antibody concentrations and titers were transformed (Log10). After confirming the transformed data were normally distributed, we calculated the geometric mean antibody titers. In this calculation, titers below the limit of the assay were assigned a value half of the lower tested dilution. Two-tailed Student’s t tests were used to compare the geometric mean antibody titers between two independent groups of mice. All statistical tests were two-tailed; probability values of less than or equal to 0.05 were considered statistically significant.

#### Ethics statement

All animal work was conducted in strict accordance with the recommendations in the Guide for the Care and Use of Laboratory Animals of the U.S. National Institutes of Health. The protocol was approved by the Children’s Hospital & Research Center at Oakland Institutional Animal Care and Use Committee (NIH assurance number A3631–01). We used approved operating protocols for vaccine injections, blood collection and euthanasia, which were performed under inhalation isoflurane anesthesia to provide minimal distress to the animals. The mice were monitored by the investigators and the animal caretakers in the Animal Facility, and were provided enrichment in the form of cotton fiber pads. Terminal blood collections were done at the earliest times possible while allowing for maximal immune responses. We used the smallest numbers of animals possible to obtain statistically meaningful data, and we did not duplicate experiments unnecessarily. The clinical isolates used to measure serum bactericidal activity were from collections described in previous studies [41], [53]. The isolates were coded and identified based only on year of isolation and country of origin. Permission to use these isolates was obtained from the Children’s Hospital Oakland Institutional Review Board (IRB). The human complement source for measuring serum bactericidal activity was serum from an adult who participated in a protocol that was approved by the IRB. Written informed consent was obtained from the subject.

## Results

### Characterization of the NOMV Vaccines

**Figure 3:**
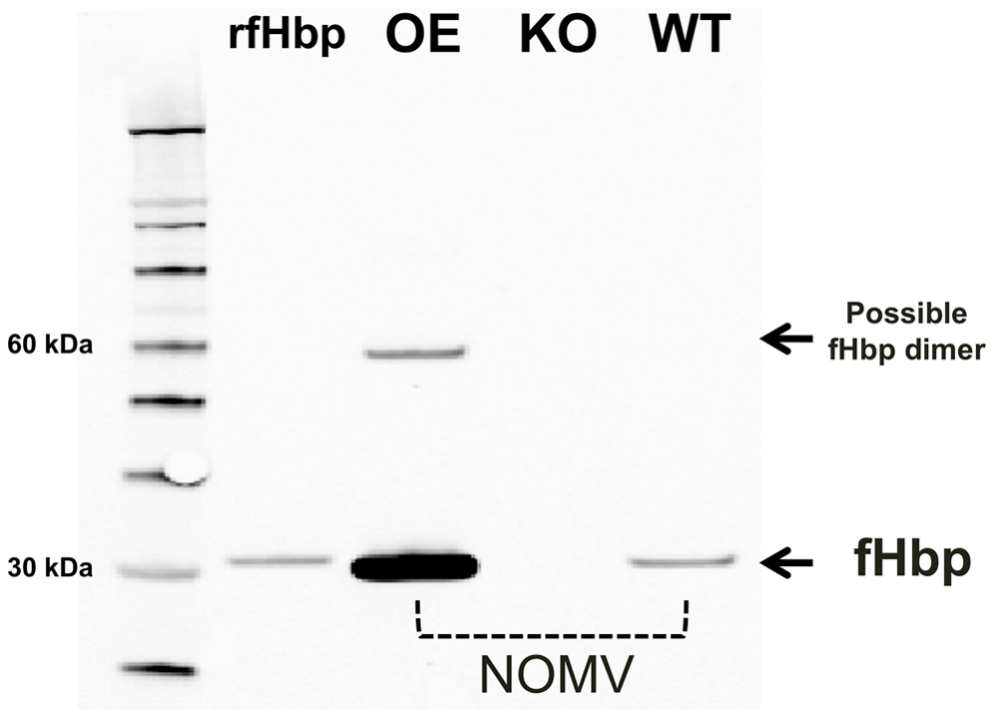
Detection of fHbp in NOMV vaccines by Western blot using anti-fHbp mAb JAR 5. rfHbp, recombinant fHbp ID 9. OE, NOMV-fHbp vaccine with over-expressed R41S mutant fHbp prepared from the mutant vaccine strain with Δ*lpxl1* and Δcapsule; KO, NOMV prepared from a triple knockout (Δ*fHbp*, Δ*lpxl1*, and Δcapsule); WT, NOMV vaccine prepared from the wildtype strain. A total of 0.25 µg of NOMV was loaded in each lane.

By SDS-PAGE, the expected major proteins in the NOMV were observed (Figure S2). Compared with the NOMV-fHbp vaccine, the NOMV-KO vaccine appeared to have slightly more protein in bands resolving around 38 and 32 kDa, which were consistent with PorB and reduction modifiable protein (Rmp), respectively. The fHbp is known to localize in the same portion of the gel as OpA/OpC proteins [62], and could not be clearly visualized. By Western blot, the amount of fHbp in the NOMV-fHbp vaccine was approximately 12-fold higher than in the control NOMV vaccine prepared from the wild-type strain (Figure 3), which was consistent with the 10-fold increase in live cells estimated by flow cytometry. Interestingly, we also detected a band of approximately 60 kDa in the NOMV-fHbp vaccine, which was consistent with the expected size of a fHbp dimer.

**Figure 4:**
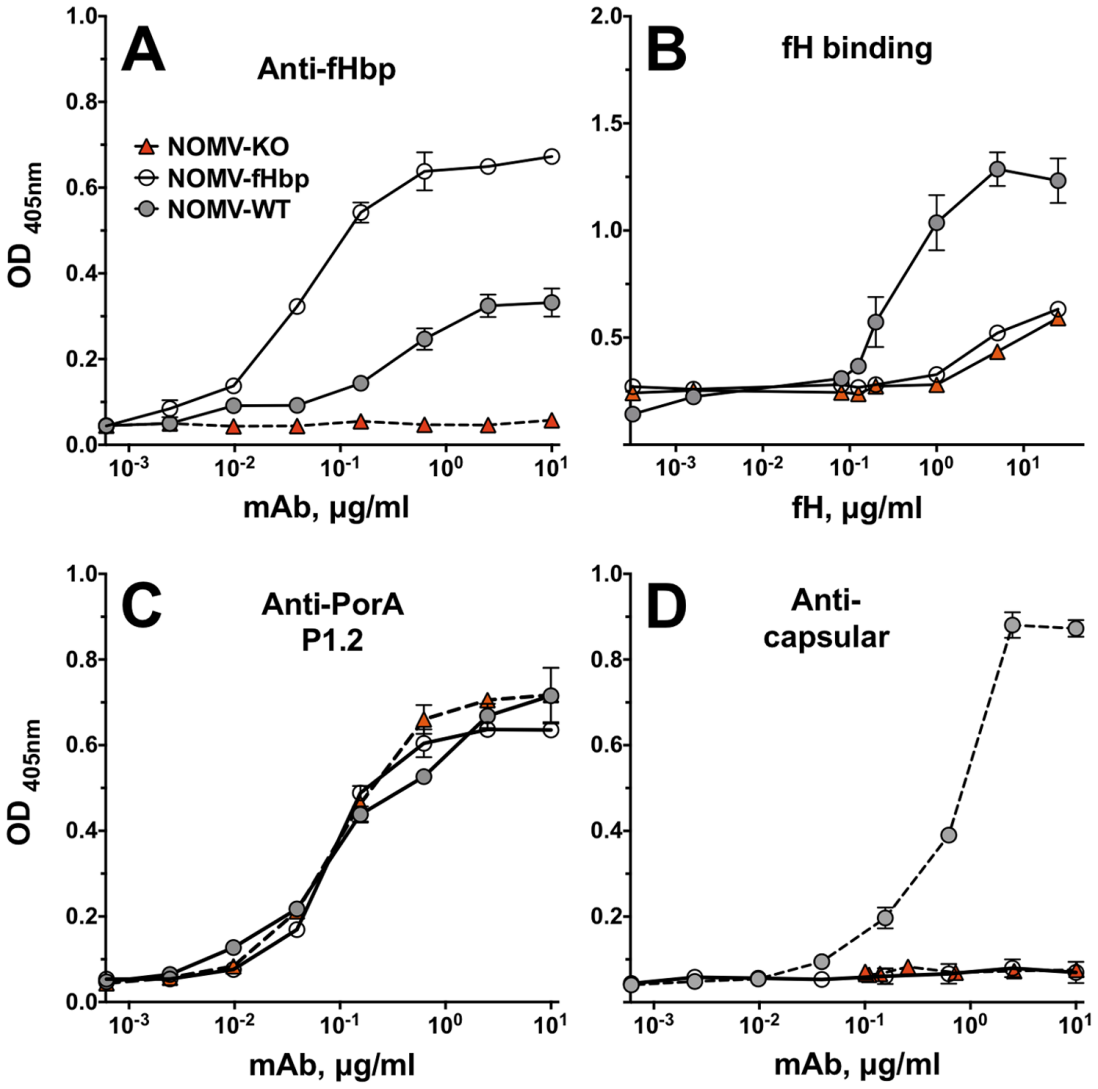
Characterization of NOMV vaccines by ELISA. Panel A. fHbp was measured using anti-fHbp mAb, JAR 5. Panel B. Binding of human fH to the NOMV vaccines was performed as previously described [43]. Panel C. PorA was measured using anti-PorA mAbs specific for P1.2 [92]. Panel D. MenW capsular polysaccharide was measured using anticapsular mAb, JW-W1.

By ELISA, the anti-fHbp mAb showed >10-fold higher binding to the NOMV-fHbp vaccine than to a control NOMV vaccine prepared from the parent wildtype strain (Figure 4, Panel A). Despite higher amounts of fHbp in the NOMV-fHbp, there was less fH binding than to the control NOMV from the wild-type strain (Panel B), which resulted from the R41S amino acid substitution in fHbp expressed by the mutant vaccine strain [45]. The similar amount of fH-binding activity by the NOMV-fHbp as the NOMV-KO vaccine likely represented residual fH binding by ligands other than fHbp (for example, NspA) [63]. Both the NOMV-fHbp and the control NOMV-KO vaccine showed similar respective binding with an anti-P1.2 PorA mAb (Panel C). As expected, the NOMV-fHbp and control NOMV-KO were negative for binding with an anticapsular mAb specific for the MenW polysaccharide, since bothe vaccines were prepared from capsule knock-out mutants (Panel E).

### The NOMV-fHbp Vaccine Elicits Broad Serum Bactericidal Activity against Epidemic Meningococcal Strains from Sub-Saharan Africa, and MenB Strains

We measured serum bactericidal antibody responses using a panel of 38 MenA, W, X and B strains. The reciprocal geometric mean titers (GMT) of two or three serum pools from mice immunized with the NOMV-fHbp or NOMV-fHbp KO vaccines against each of the strains are summarized in Table 1.

**Figure 5:**
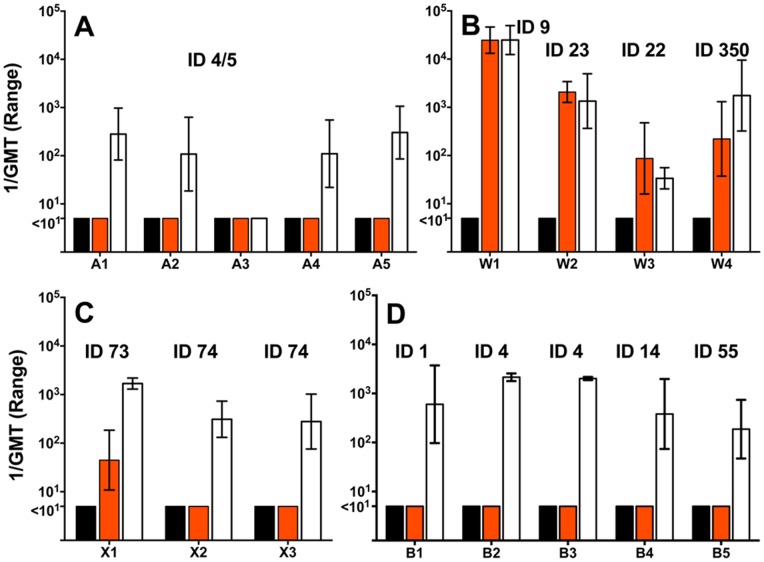
Serum bactericidal antibody responses of mice immunized with NOMV-fHbp vaccine. Panels A, B and C, MenA, W or X isolates, respectively, from cases of disease isolated during epidemics in sub-Saharan Africa. Panel D, MenB isolates from industrialized countries. The MenA, X and B strains had fHbp amino acid sequence variants in variant group 1 (See Table 1). The MenW stains had fHbp in variant group 1 (ID 9, ID 350) or variant group 2 (ID 23, ID 22). Bars represent the reciprocal GMT and ranges of three serum pools per vaccine group, and two pools for the adjuvant alone (four individual sera in each pool). Black bars, control mice immunized with aluminum hydroxide without a vaccine antigen; orange bars, control mice immunized with a NOMV-fHbp KO vaccine; white bars, mice immunized with the NOMV-fHbp vaccine.

Mice immunized with the NOMV-fHbp vaccine alone developed serum bactericidal titers >1∶10 against 13 of 15 African MenA strains with fHbp ID 4 or 5 in variant group 1; 10 out of 10 African MenX strains with fHbp ID 73 or 74 in variant group 1, and 7 of 7 African MenW strains with fHbp ID 9, 23, or 349 in variant groups 1, 2, or 3 respectively. The NOMV-fHbp also elicited serum bactericidal titers >1∶10 against all six MenB strains tested from the US, Norway, New Zealand or The Gambia. All of these MenB strains were selected to have fHbp in variant group 1 (ID 1, 4, 14, 55 or 347) to match the variant group of the fHbp ID 9 in the NOMV-fHbp vaccine. For the susceptible MenA, W, X or B strains the GMT usually was >1∶100. The two exceptions were a MenA strain (A11, GMT 1∶71) and a MenW strain (W3, GMT 34). For representative strains tested with 3 serum pools per vaccine group, the reciprocal geometric mean titers (GMT) and ranges of the three titers are shown in Figure 5.

The MenA and B strains had heterologous PorA VR types to the P1.5,2 in the NOMV vaccines. The titers elicited by the control NOMV-KO vaccine against these strains generally were <1∶10 (Figure 5 and Table 1). One notable exception was a MenA strain (A12) with a GMT of 1∶632 elicited by the NOMV-KO vaccine (Table 1), and 1∶1643 by the NOMV-fHbp vaccine; the antigenic target of the bactericidal antibodies elicited by the NOMV-KO vaccine was not identified. The four MenX strains with PorA 5-1,10-1, which was anticipated to be antigenically related to PorA P1.2,5 in the vaccine, were either resistant (N = 2 isolates, titers <1∶10), or only moderately susceptible to antibodies elicited by the NOMV-KO vaccine (N = 2 isolates, bactericidal titers of 1∶38 and 1∶93).

Against all 7 MenW test strains, there were no significant differences in bactericidal responses elicited by NOMV-fHbp or control NOMV-KO vaccines (Figure S3, respective GMTs of 1053 vs. 1139, P>0.8). For the five MenW strains with a homologous PorA VR type of P1.5,2 to the vaccine, the GMTs elicited by the NOMV-KO vaccine were >1000, which were within one-dilution of those elicited by the NOMV-fHbp vaccine. Two MenW strains had PorA VR types of P1.5–1,2–36 (W3) or P1.5–1,2–2 (W4), which were related to P1.5,2 in the vaccine. The GMTs elicited by the NOMV-KO vaccine against these strains were 1∶87 and 1∶221, respectively, compared to 1∶34 and 1∶1753 for the NOMV-fHbp vaccine (Figure 5). The higher GMT elicited by the NOMV-fHbp vaccine against strain W4 can be explained by the presence of fHbp ID 350 in variant group 1, which was similar to the one in the vaccine (ID 9), while the lower GMT against strain W3 reflected fHbp ID 22 in variant group 2.

### The Combination NOMV-fHbp/MenA Conjugate Vaccine Elicits Enhanced IgG Antibody Responses and Enhanced Serum Bactericidal Activity

We used the liquid NOMV-fHbp that had been adsorbed with aluminum hydroxide to reconstitute a lyophilized MenA conjugate vaccine from Novartis (See methods). Control mice received the NOMV-fHbp vaccine alone, a European/U.S.-licensed quadrivalent A,C,Y,W conjugate vaccine (MCV4-CRM, Novartis), a NOMV-fHbp KO vaccine, or aluminum hydroxide without a vaccine antigen. For the combination NOMV-fHbp/MenA vaccine study, we measured titers in sera from individual mice to increase statistical power to detect significant group differences in the responses, if they were present.

**Figure 6:**
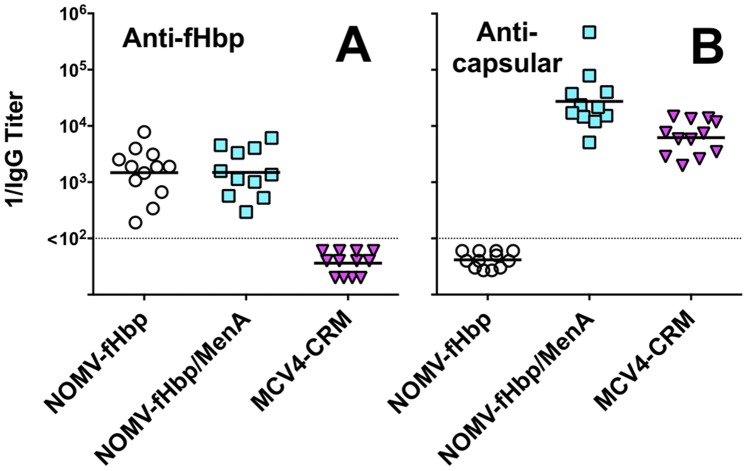
Serum IgG antibody responses to vaccination as measured by ELISA. Panel A. Anti-fHbp ID 9 titers. Panel B. Anticapsular antibody titers to MenA polysaccharide. Each symbol represents the reciprocal titer of an individual immunized mouse. Horizontal lines denote reciprocal geometric mean titers. There were no significant differences in the IgG anti-fHbp antibody titers elicited by the NOMV-fHbp or combination NOMV-fHbp/MenA conjugate vaccine (P>0.5). The serum IgG anticapsular titers were higher in mice immunized with the combination NOMV-fHbp/Men A conjugate vaccine than the control MCV4-CRM conjugate vaccine (P = 0.001).

There were no significant differences between the serum IgG anti-fHbp titers of mice immunized with the combination NOMV-fHbp/MenA conjugate vaccine or the NOMV-fHbp vaccine alone (Figure 6, Panel A). Mice immunized with the combination vaccine had four-fold higher serum IgG group A anticapsular antibody titers than mice immunized with the control MCV4-CRM conjugate vaccine (P = 0.001, Panel B). Although we did not include a control group of mice immunized with the MenA conjugate component alone, the data suggested that there was no impairment of the group A anticapsular antibody responses elicited by the combination NOMV-fHbp/MenA conjugate vaccine.

**Figure 7:**
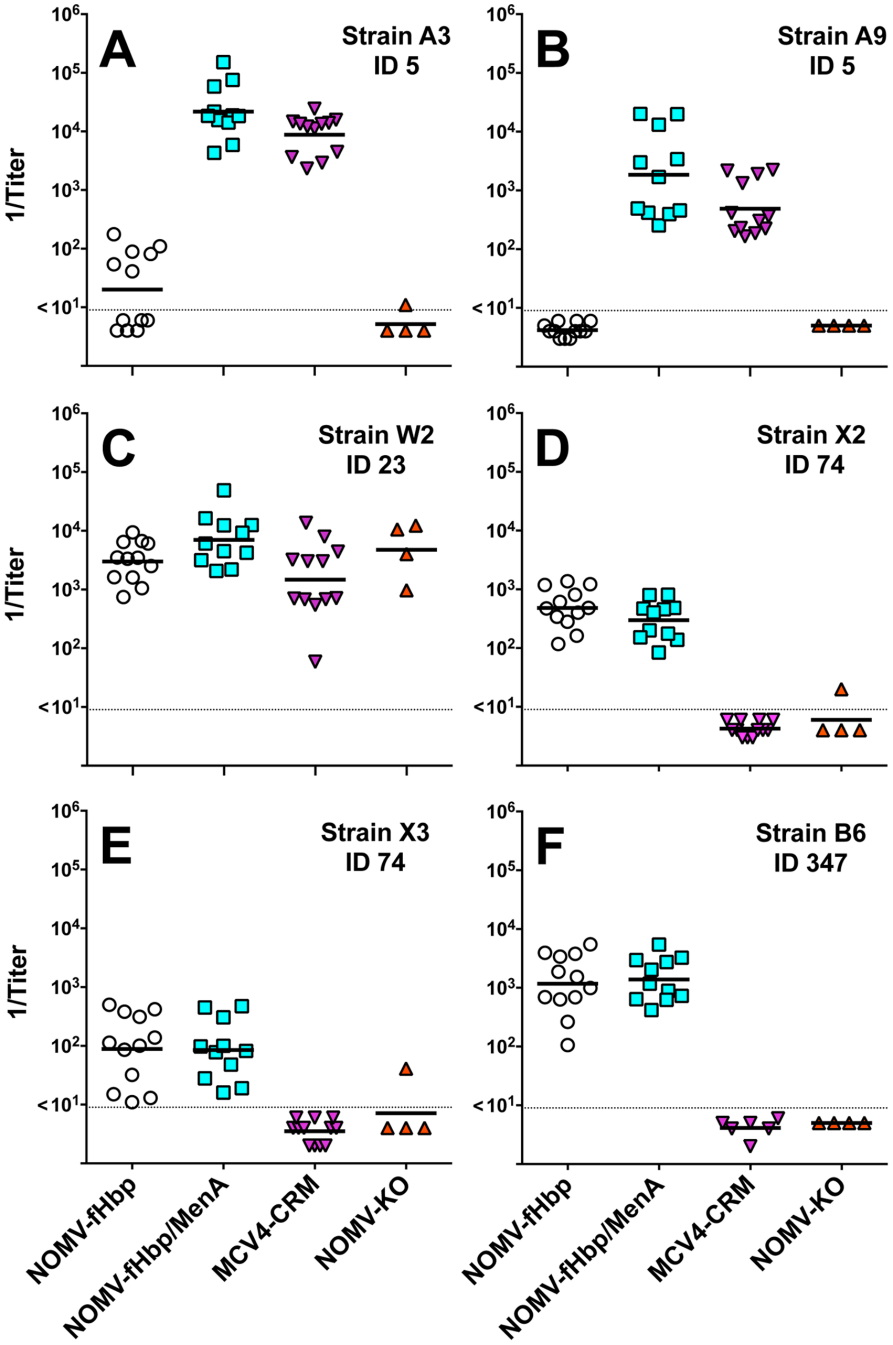
Serum bactericidal antibody responses of mice immunized with a combination NOMV-fHbp/MenA conjugate vaccine. Test strains are described in Table 1, and strain selection criteria are described in the text. Panels A and B, MenA strains A3 and A9 with fHbp ID 5, respectively in variant group 1. Panel C, MenW strain, W2, with fHbp ID 23 in variant group 2. Panels D and E, MenX strains, X2 or X3, respectively, with fHbp ID 74 in variant group 1. Panel F, MenB strain, B6, from The Gambia, with fHbp ID 347 in variant group 1. Each symbol represents the reciprocal titer of an individual mouse; horizontal bars represent 1/GMT. The respective serum bactericidal titers elicited by the combination NOMV-fHbp/MenA vaccine were higher than the NOMV-fHbp alone against strains A3 and A9 (P<0.0001) and strain W2 (P = 0.03). For these three strains, the serum bactericidal titers elicited by the combination NOMV-fHbp/MenA conjugate vaccine also were higher than the respective titers elicited by control MCV4-CRM vaccine (P<0.03). For the two MenX strains and the MenB strain, the respective titers elicited by the combination NOMV-fHbp/MenA conjugate vaccine were not significantly different from those elicited by the NOMV-fHbp vaccine alone (P>0.14).

We chose strains A3 and A9 to assess MenA bactericidal responses because both strains were resistant to bactericidal activity of antibodies elicited by the NOMV-fHbp vaccine alone (GMT <1∶10, Table 1). For MenW, we chose strain W2 with fHbp ID 23 in variant group 2. Since the fHbp of this strain was in a different variant group than the variant group 1 fHbp antigen in the NOMV-fHbp vaccine, the bactericidal antibody responses largely reflected antibodies to non-fHbp antigens in the NOMV (the largest contributor likely being PorA). The serum bactericidal titers elicited by the combination NOMV-fHbp/MenA conjugate vaccine against these MenA or W strains were higher than to the NOMV-fHbp alone. (Figure 7, Panels A, B, and C, P<0.03). The serum bactericidal titers elicited by the combination NOMV-fHbp/MenA conjugate vaccine also were higher than the respective titers elicited by the control MCV4-CRM conjugate vaccine (P<0.03).

We also measured serum bactericidal titers against two representative MenX strains with fHbp ID 74 in variant group 1 (X2 and X3, Panels D and E, respectively), and a MenB strain from The Gambia with fHbp ID 347 in variant group 1 (B6, Panel F). These three strains had heterologous PorA to the vaccine strain and the serum bactericidal activity largely reflected antibody to fHbp. For all three strains, the respective titers elicited by the combination vaccine were not significantly different from the NOMV-fHbp vaccine alone P>0.14). As expected, none of the MenX or MenB strains was killed by complement together with sera from control mice immunized with the MCV4-CRM conjugate vaccine.

## Discussion

The MenA conjugate vaccine, MenAfriVac, introduced in sub-Sahara is safe and highly immunogenic [9] but coverage does not include MenW or MenX strains, which cause epidemics in this region [13]–[15]. One approach to expand protection to these serogroups would be a multivalent polysaccharide-protein conjugate vaccine, which also might include MenC and/or MenY. The technical feasibility of developing a multivalent meningococcal conjugate vaccine has been demonstrated by commercially available quadrivalent MenA,C,Y,W conjugate vaccines [16]–[19]. Vaccine grade MenX polysaccharide has been characterized [64], and favorable preliminary data on experimental MenX polysaccharide-protein conjugate vaccines have been presented (Romano, MR et al. Poster 150, XVIIIth International Pathogenic Neisseria Conference. Würzburg, Germany, September 2012, http://www.conventus.de/fileadmin/media/2012/ipnc/IPNC2012_Programme_and_Abstractbook_web.pdf). The cost of production, of a multivalent conjugate vaccine that includes MenA, W and X and, possibly, additional polysaccharides, is likely to be higher than the 1 US dollar per dose price point, which was considered essential for having a sustainable meningococcal vaccine immunization program in sub-Sahara [5], [65]. Also, the technical challenges required for development and licensure of a multivalent conjugate vaccine for Africa are greater than for a monovalent MenA conjugate vaccine. Specifically there is a high risk that one of the components of the multivalent conjugate will have suboptimal immunogenicity and require adjustment of dosage or formulation [66]–[68]. There also may be interference in immunogenicity between the individual components of the vaccine (the more components, the higher the risk; for a review please see [69]). With a multivalent conjugate vaccine there also is a risk that strains with serogroups not contained in the vaccine (for example, MenB), might emerge in Africa and cause epidemics. While the time required for development and licensure of a NOMV-fHbp/MenA conjugate vaccine also will be long, and the regulatory issues will be challenging, the uncertainties accompanying development of a multivalent conjugate vaccine underscore the need to investigate alternative vaccine approaches for preventing non-MenA epidemics in Africa.

Conventional detergent-treated outer membrane vesicle (dOMV) vaccines have been developed for prevention of MenB disease. The detergent treatment is used to decrease endotoxin activity but also extracts desirable antigens such fHbp [36]. dOMV vaccines have been given safely to millions of children and young adults [70], and have been demonstrated to be effective for control of epidemics caused by MenB strains [71], [72]. Recent pre-clinical studies also demonstrated the feasibility of developing a dOMV vaccine for prevention of MenA and MenW disease [73]. A major limitation of dOMV vaccines, however, is that the protective antibodies in infants and childrens are largely directed against PorA [74], which is antigenically variable [75], [76]. A dOMV vaccine for prevention of the main epidemic clones in the meningitis belt will need to include PorA antigens from prevalent MenA, MenW and MenX strains. While the PorA VR types of some of these clones (such as MenA) have been stable over the last decade [46], [77]–[79], dOMV vaccines likely will require frequent substitutions of the vaccine strains as the PorA of emerging strains changes over-time [80].

An important difference between NOMV and dOMV vaccines is that endotoxin activity of NOMV vaccines is decreased genetically by inactivating a gene (*lpxL1*) in lipid biosynthesis, which avoids the need for detergent treatment of the vesicles in the vaccine. In a phase 1 study in adults, a prototype meningococcal NOMV vaccine prepared from an *lpxL1* knock-out strain appeared to be well tolerated [81]. The vaccine strain used to prepare an NOMV vaccine also can contain additional genetic modifications to increase expression of desirable antigens to enhance induction of protective antibodies, or deletion of unwanted molecules to increase safety, and/or lower to production costs (for example, by inactivating *gna33*, which increases release of vesicles from the bacteria [82]). NOMV vaccines also can elicit protective antibodies against more than one antigenic target, which may delay emergence of escape mutants.

In the present study, the NOMV-fHbp vaccine prepared from the mutant strain elicited broad serum bactericidal activity against a panel of Africa epidemic MenA, W and X strains and genetically diverse MenB strains with fHbp in variant group 1 from Norway, New Zealand, the U.S. and The Gambia. Of the 38 strains tested, two MenA strains were resistant to bactericidal activity of the NOMV-fHbp antisera (GMT <1∶10). By flow cytometry, the resistant strains had similar fHbp expression levels as MenA strains susceptible to anti-fHbp bactericidal activity (unpublished data). The basis of the resistance is therefore not known and is currently being investigated. This observation, however, together with our long-term goal to build upon the recent successful introduction of a MenA conjugate vaccine in Africa, suggested that combining the NOMV-fHbp vaccine with a MenA conjugate vaccine could augment protection against MenA strains. In mice, the resulting combination NOMV-fHbp/MenA conjugate vaccine elicited serum antibodies with greater bactericidal activity against MenA and W strains than the control MCV4-CRM vaccine. The combination NOMV-fHbp/MenA conjugate vaccine also provided coverage against MenX and B strains, which were not covered by the licensed meningococcal conjugate vaccine.

The reasons for the higher serum bactericidal antibody responses against the serogroup A and W strains in the NOMV-fHbp/MenA conjugate vaccine group, compared with the MCV4-CRM vaccine group, are not known. Conceivably, natural adjuvants in the NOMV-fHbp component such as PorB [83], [84] or LOS [49], [85] contributed to the higher antibody responses. An additional possibility is that anti-fHbp antibodies elicited by the NOMV-fHbp vaccine component acted synergistically with MenA anticapsular antibodies elicited by the MenA conjugate and resulted in higher MenA bactericidal activity than with either antibody individually [86]. Further studies are needed to elucidate the mechanism. Collectively, our results demonstrate that a combined NOMV-fHbp/MenA conjugate vaccine can provide broad coverage against African MenA, X and W strains, which are the principal causes of epidemics in the region, and also against MenB strains. Most likely the NOMV-fHbp/MenA vaccine would require two or three doses for maximum protection, which is compatible for routine vaccination of infants and toddlers, age groups at high risk for developing meningococcal disease. During epidemics, a single dose of the combination vaccine also could be used to boost immunity in older children or adults previously given the vaccine.

### Limitations of the Study

The African continent is vast, and the epidemiology of meningococcal disease is complex with major seasonal and temporal changes in incidence rates, and geographical differences in strain prevalence, which remain poorly understood [1], [46], [77], [87], [88]. Further, the number of isolates investigated from different epidemics represents only a small fraction of the total number of cases [46]. Our previous data from studies of MenA strains collected from epidemics spanning 50 years indicated that only two fHbp variants, ID 4 or 5, which differed from each other by only one amino acid, were present in all isolates. The available data, however, on fHbp sequence variants of MenX and MenW isolates from Africa are more limited and it is possible that there is greater fHbp diversity among strains with these serogroups than MenA strains. Thus, for a broadly protective NOMV-fHbp vaccine intended for all countries in sub-Sahara, additional strain characterization and epidemiology data are needed, particularly for MenX and MenW strains. It is also is possible that additional fHbp sequence variants and/or other antigens might be needed in the NOMV vaccine to ensure optimal coverage, particularly against MenB strains with fHbp in variant groups 2 or 3, should serogroup B strains emerge to cause epidemics in Africa.

A second limitation of our study was the small number of isolates tested for bactericidal activity using sera from individual mice immunized with the NOMV-fHbp/MenA conjugate vaccine (Figure 7). However, we tested susceptibility of a much larger strain panel using pooled serum from immunized mice (33 isolates from 13 different Sub-Saharan countries, and South Africa, including 8 different clonal complexes, Table 1). All but two of the group A strains were susceptible to bactericidal activity of pooled antisera from mice immunized with the NOMV-fHbp vaccine. Further, these two serogroup A strains, A3 and A9, were tested for bactericidal activity of sera from individual mice immunized with the NOMV-fHbp/MenA conjugate combination vaccine and were highly susceptible.

A third limitation of our study is lack of information on whether or not NOMV-fHbp immunization will decrease asymptomatic carriage and transmission of the *N. meningitidis* in the population. One of the strengths of polysaccharide conjugate vaccines is their ability to induce herd immunity by decreasing asymptomatic carriage and transmission in the population [12], [89]. This question is important for an NOMV-fHbp vaccine for Africa, but is difficult to investigate experimentally in mice because receptors that are important for nasopharyngeal colonization of *N. meningitidis*, such as CEACAM, are human specific [90], [91].

For testing proof of principal of a combination NOMV-fHbp/MenA conjugate vaccine, we used a lyophilized MenA conjugate vaccine from Novartis, which is likely to be too expensive for inclusion in a vaccine intended for Africa. The feasibility of combining the NOMV-fHbp vaccine with the lyophilized MenA conjugate vaccine being used in Africa (MenAfriVac), therefore should be investigated. For our study with the Novartis MCV4-CRM conjugate vaccine we used one-tenth of the human dose in the mice. Although this dose elicited high titers of serum anticapsular antibodies (Figure 6), the dose may not have been optimally immunogenic. For this reason the superior serogroup A and W bactericidal antibody responses elicited by the NOMV-fHbp/MenA conjugate vaccine when compared to the MCV4-CRM vaccine, needs to be interpreted with caution.

The cost of production of an NOMV-fHbp vaccine currently is uncertain and will depend on the growth characteristics of the final mutant strain selected and large scale manufacturing processes that will need to be developed. Low cost dOMV-based vaccines, however, have been manufactured in Cuba and Brazil, and the processes used are within the technical abilities of developing country manufacturers. Finally, a vaccine intended for Africa must be conveniently administered and stable under stressful environmental conditions. Our studies did not investigate these questions, which will require additional studies. Despite these limitations, the excellent breadth of protective antibodies elicited in mice by the prototype NOMV-fHbp/MenA conjugate vaccine investigated in the present study provide ample justification for conducting additional studies to address these technical questions as well as advancing the vaccine for testing proof of principle in a human vaccine trial.

## Supporting Information

Figure S1 **Network analysis of prominent individual fHbp sequence variants of meningococcal isolates from Africa.** The analysis was generated using SplitsTree, version 4.0 (http://www.splitstree.org/). Data shown are for the five predominant fHbp sequence variants and two related sequences with one amino acid differences, from 124 fHbp African isolates investigated in a previous study [42]. The network, using the hamming distance, represents how similar (closer in the network) or different (far) are the fHbp sequences present among African isolates. The scale bar refers to 0.01 differences per unit of length. Numbers represent the specific fHbp ID designation for each sequence. ID 4 and 5 and found among serogroup A isolates; ID 73 and ID 74 are found among serogroup X isolates, and ID 9, ID 22 and ID 23 are found among serogroup W isolates. Note that fHbp ID 4 and ID 5 differ from each other by one amino acid, and fHbp ID 22 and ID 23 differ from each other by one amino acid.
(DOCX)

Figure S2 **Detection of major proteins in NOMV vaccines.** Major proteins in NOMV vaccines as visualized by Coomassie-stained SDS-PAGE. rfHbp, recombinant fHbp ID 9 control. OE, NOMV-fHbp vaccine with over-expressed R41S mutant fHbp prepared from the mutant vaccine strain with Δ*lpxl1* and Δcapsule; KO, NOMV prepared from a triple knockout (Δ*fHbp*, Δ*lpxl1*, and Δcapsule). A total of 5 µg of NOMV was loaded in each lane, and 0.25 µg of the recombinant fHbp.
(DOCX)

Figure S3 **Comparison of serum bactericidal antibody responses of mice immunized with NOMV-fHbp or NOMV-fHbp KO vaccines.** Serum bactericidal antibody responses were measured against seven serogroup W isolates with porA VR types related to the PorA contained in both NOMV vaccines. The antibody responses elicited by the NOMV-fHbp KO vaccine are mostly directed at PorA. Bars represent the reciprocal GMT of two or three serum pools. Orange bars, control mice immunized with an NOMV vaccine prepared from the mutant vaccine strain with Δ*lpxl1* and Δcapsule in which the gene for fHbp had been inactivated; white bars, mice immunized with the NOMV vaccine with Δ*lpxl1* and Δcapsule and over-expressed fHbp. There were no significant differences in bactericidal responses elicited by two vaccines (respective GMTs of 1053 vs. 1139, P>0.8 by paired T test).
(DOCX)
