# Supplementary material for: Mutant Native Outer Membrane Vesicles Combined with a Serogroup A Polysaccharide Conjugate Vaccine for Prevention of Meningococcal Epidemics in Africa
Source: PLoS One. 2013 Jun 21;8(6):e66536. doi: 10.1371/journal.pone.0066536 (PMC3689835; doi:10.1371/journal.pone.0066536)
Supplement: Figure S1 — Network analysis of prominent individual fHbp sequence variants of meningococcal isolates from Africa. The analysis was generated using SplitsTree, version 4.0 (http://www.splitstree.org/). Data shown are for the five predominant fHbp sequence variants and two related sequences with one amino acid differences, from 124 fHbp African isolates investigated in a previous study [42]. The network, using the hamming distance, represents how similar (closer in the network) or different (far) are the fHbp sequences present among African isolates. The scale bar refers to 0.01 differences per unit of length. Numbers represent the specific fHbp ID designation for each sequence. ID 4 and 5 and found among serogroup A isolates; ID 73 and ID 74 are found among serogroup X isolates, and ID 9, ID 22 and ID 23 are found among serogroup W isolates. Note that fHbp ID 4 and ID 5 differ from each other by one amino acid, and fHbp ID 22 and ID 23 differ from each other by one amino acid. (DOCX) [file pone.0066536.s001.docx]

**Supplementary Figure S1:** Network analysis of prominent individual fHbp sequence variants

Network analysis of prominent individual fHbp sequence variants of meningococcal isolates from Africa. The analysis was generated using SplitsTree, version 4.0 (http://www.splitstree.org/). Data shown are for the five predominant fHbp sequence variants and two related sequences with one amino acid differences, from 124 fHbp African isolates investigated in a previous study [42]. The network, using the hamming distance, represents how similar (closer in the network) or different (far) are the fHbp sequences present among African isolates. The scale bar refers to 0.01 differences per unit of length. Numbers represent the specific fHbp ID designation for each sequence. ID 4 and 5 and found among serogroup A isolates; ID 73 and ID 74 are found among serogroup X isolates, and ID 9, ID 22 and ID 23 are found among serogroup W isolates. Note that fHbp ID 4 and ID 5 differ from each other by one amino acid, and fHbp ID 22 and ID 23 differ from each other by one amino acid..
