# Supplementary material for: Mutant Native Outer Membrane Vesicles Combined with a Serogroup A Polysaccharide Conjugate Vaccine for Prevention of Meningococcal Epidemics in Africa
Source: PLoS One. 2013 Jun 21;8(6):e66536. doi: 10.1371/journal.pone.0066536 (PMC3689835; doi:10.1371/journal.pone.0066536)
Supplement: Figure S2 — Detection of major proteins in NOMV vaccines. Major proteins in NOMV vaccines as visualized by Coomassie-stained SDS-PAGE. rfHbp, recombinant fHbp ID 9 control. OE, NOMV-fHbp vaccine with over-expressed R41S mutant fHbp prepared from the mutant vaccine strain with Δlpxl1 and Δcapsule; KO, NOMV prepared from a triple knockout (ΔfHbp, Δlpxl1, and Δcapsule). A total of 5 µg of NOMV was loaded in each lane, and 0.25 µg of the recombinant fHbp. (DOCX) [file pone.0066536.s002.docx]

**Figure S2:** Major proteins in NOMV vaccines

Major proteins in NOMV vaccines as visualized by Coomassie-stained SDS-PAGE. rfHbp, recombinant fHbp ID 9 control. OE, NOMV-fHbp vaccine with over-expressed R41S mutant fHbp prepared from the mutant vaccine strain with Δ*lpxl1* and Δcapsule; KO, NOMV prepared from a triple knockout (Δ*fHbp*, Δ*lpxl1*, and Δcapsule). A total of 5 µg of NOMV was loaded in each lane, and 0.25 µg of the recombinant fHbp.
