# Supplementary material for: Mutant Native Outer Membrane Vesicles Combined with a Serogroup A Polysaccharide Conjugate Vaccine for Prevention of Meningococcal Epidemics in Africa
Source: PLoS One. 2013 Jun 21;8(6):e66536. doi: 10.1371/journal.pone.0066536 (PMC3689835; doi:10.1371/journal.pone.0066536)
Supplement: Figure S3 — Comparison of serum bactericidal antibody responses of mice immunized with NOMV-fHbp or NOMV-fHbp KO vaccines. Serum bactericidal antibody responses were measured against seven serogroup W isolates with porA VR types related to the PorA contained in both NOMV vaccines. The antibody responses elicited by the NOMV-fHbp KO vaccine are mostly directed at PorA. Bars represent the reciprocal GMT of two or three serum pools. Orange bars, control mice immunized with an NOMV vaccine prepared from the mutant vaccine strain with Δlpxl1 and Δcapsule in which the gene for fHbp had been inactivated; white bars, mice immunized with the NOMV vaccine with Δlpxl1 and Δcapsule and over-expressed fHbp. There were no significant differences in bactericidal responses elicited by two vaccines (respective GMTs of 1053 vs. 1139, P>0.8 by paired T test). (DOCX) [file pone.0066536.s003.docx]

**Supplementary Figure S3**. Comparison of serum bactericidal antibody responses of mice immunized with NOMV-fHbp or NOMV-fHbp KO vaccines.

Serum bactericidal antibody responses were measured against seven serogroup W isolates with porA VR types related to the PorA contained in both NOMV vaccines. The antibody responses elicited by the NOMV-fHbp KO vaccine are mostly directed at PorA. Bars represent the reciprocal GMT of two or three serum pools. Orange bars, control mice immunized with an NOMV vaccine prepared from the mutant vaccine strain with Δ*lpxl1* and Δcapsule in which the gene for fHbp had been inactivated; white bars, mice immunized with the NOMV vaccine with Δ*lpxl1* and Δcapsule and over-expressed fHbp. There were no significant differences in bactericidal responses elicited by two vaccines (respective GMTs of 1053 vs. 1139, P>0.8 by paired T test).
